# Prediction of the Prevalence of Hypertension and Associated Risk Factors in Rwanda Using Gibbs Sampling Method

**DOI:** 10.3390/diseases11020087

**Published:** 2023-06-16

**Authors:** Angélique Dukunde, Jean Marie Ntaganda, Juma Kasozi, Joseph Nzabanita

**Affiliations:** 1 African Center of Excellence in Data Science-Biostatistics, College of Business and Economics, University of Rwanda, Kigali P.O. Box 4285, Rwanda; 2 Department of Mathematics, College of Science and Technology, University of Rwanda, KN 67 Street, Nyarugenge P.O. Box 3900, Rwanda; 3 Department of Mathematics, College of Natural Science, Makerere University, Kampala P.O. Box 7062, Uganda

**Keywords:** non-communicable disease, hypertension, prediction, Gibbs sampling method, Markov Chain Monte Carlo

## Abstract

In Rwanda, the prevalence of hypertension was 15.3% in 2015. At present, there are no accurate predictions of the prevalence of hypertension and its trend over time in Rwanda to assist decision makers in making plans for prevention and more effective interventions. This study used the Gibbs sampling method in combination with the Markov Chain Monte Carlo approach to predict the prevalence of hypertension and its associated risk factors in Rwanda over a period of ten years. The data were from World Health Organization (WHO) reports. The findings showed that the prevalence of hypertension is estimated to reach 17.82% in 2025, with tobacco use, being overweight or obese, and other risk factors having a respective prevalence of 26.26%, 17.13%, 4.80%, and 33.99%, which shows the increase and, therefore, measures for prevention to be taken. Therefore, to prevent and reduce the prevalence of this disease, the government of Rwanda should take appropriate measures to promote a balanced diet and physical exercise.

## 1. Introduction

The best-known non-communicable diseases (NCDs) are cancer, diabetes, cardiovascular diseases, and chronic respiratory diseases. These are responsible for disability and have a high rate of death worldwide [1].

Hypertension, commonly referred to as systolic blood pressure (SBP), is a condition that occurs when blood pressure is more than 140 mmHg. There are four stages of hypertension: normal blood pressure with pressure below 120/80 mmHg, high–normal blood pressure with a systolic pressure ranging from 120 to 129 mmHg and a diastolic pressure below (not above) 80 mmHg; Grade 1 hypertension with a systolic pressure ranging from 130 to 139 mmHg or a diastolic pressure ranging from 80 to 89 mmHg; and Grade 2 hypertension with a systolic pressure of greater than 140/90 mmHg [2,3].

Uncontrolled hypertension can result in complications such as heart failure, kidney failure, angina, memory loss, stroke, and cardiovascular problems. Hypertension is referred to as a “silent killer disease” because no noticeable symptoms can be seen until major complications and consequences arise. Risk factors for hypertension include smoking, passive smoking exposure, diabetes, obesity, high cholesterol, unhealthy diets, and physical inactivity. These factors collectively increase the risk of acquiring high blood pressure (HBP) [4]. The worst hypertension complication is artery damage, which causes blood vessels to narrow and requires the heart to work harder to pump blood to the body [5].

At least 90% of people fall into the category of essential or primary hypertension, which is a type of hypertension that could arise from unknown causes and is treatable but incurable. Blood plasma volume and the activity of the hormones that control blood volume and pressure may be the primary causes of primary hypertension. If this is not the case, it is referred to as secondary hypertension, which has specific causes as a side effect of other conditions or disease processes, such as diabetes and its effects, kidney disease, a rare adrenal gland cancer, corticosteroids, congenital adrenal hyperplasia, a disorder of the cortisol-secreting adrenal glands, an overactive thyroid gland, or the level of calcium and phosphorus [4,6].

Family history, being overweight, poor diet, too much salt, drinking too much alcohol, not exercising, cigarette smoking, and kidney problems are various and serious causes of hypertension [7]. The good and interesting thing about hypertension is that it can be prevented [8].

High blood pressure leads to narrowed, weakened, and hardened arteries, which cannot deliver blood efficiently to the kidneys [9].

A total of 71% (41 million) of total deaths (51 million) worldwide are due to NCDs and injuries, mainly comprising cardiovascular diseases (31%), cancers (16%), chronic respiratory diseases (7%), and diabetes (3%) [10].

The African region has the highest prevalence of hypertension among adults over 25 years of age, causing a massive economic burden for the continent, including the cost of caring for all the complications arising from hypertension, such as cerebrovascular disease, ischemic heart disease, and congestive heart failure, as well as indirect costs such as decreases in the productivity of workers struck by stroke, heart failure, and ischemic heart disease [11].

Research indicated that blood vessels naturally harden with age, losing their elasticity. This may be one explanation for why older people are more at risk of developing high blood pressure. Studies showed that an increase in body mass index, as well as an increase in age, are linked to increases in blood pressure and can lead to hypertension [8].

According to the authors of [6,12], 26% of people worldwide are estimated to have hypertension, and because of increased economic development, that rate is expected to rise to 29% by the year 2025. People’s lifestyles were impacted by urbanization, and research shows that hypertension is increasingly evolving into a severe public health problem in Africa. The frequency was estimated to be 30.8% in 2010, despite a substantial increase in some parts of Africa of between 36.2% and 77.3% [12,13]. In this continent, there were 92.3 million cases of hypertension in 2000, 130.2 million cases in 2010 and, according to predictions, there will be 216.8 million cases in 2030. Due to a lack of knowledge regarding the disease’s prevention, this is an extremely high rate of growth. A very low level of awareness of hypertension was discovered in Africa, ranging from 29.2% to 33.7% between 2000 and 2010, respectively [14]. Research conducted in Africa showed that hypertension is particularly common in certain African countries.

The highest rates, which ranged from 77.3% to 65.4% in 2010, were observed in South Africa, Tanzania, Tunisia, and Senegal. The prevalence rate was 33.3% in Northern Africa, 27.8% in Sub-Saharan Africa overall, 34.6% in Southern Africa, 27.3% in Western Africa, 27.1% in Central Africa, and 26.7% in Eastern Africa [15]. Researchers found that Tanzania has a high pre-hypertension rate of 36.2% but a lower prevalence of hypertension (8.0%) [16]. According to [17], researchers found that South Africa had an extremely high prevalence of hypertension, at 49.2%.

This study, conducted in Rwanda, is relevant to all of Africa as well as middle-income countries because the prevalence of hypertension in Africa varies from region to region. It is crucial to regularly carry out extensive research on the disease in Rwanda and other African nations because the prevalence of hypertension in Africa varies from region to region. This will help medical practitioners to make an early, accurate diagnosis by helping them to comprehend the components of hypertension screening.

According to a population-based study on the risk factors for non-communicable diseases (NCDs) using the World Health Organization’s (WHO’s) approach [18], the prevalence of hypertension was 15.3% in Rwanda in 2015. This study employed a descriptive cross-sectional technique to test blood pressure and ask respondents about variables related to screening uptake in Kanjongo, Nyamasheke District, Rwanda, where the prevalence of hypertension was 17.5% [13]. 

Based on Rwandan Health Management Information Systems’ (HMIS’) data, covering the period of January–December 2013 and focusing on the top eight causes of morbidity in district hospitals, 2013, NCDs accounted for at least 51.86% of all the district hospital outpatient’s consultations and 22.3% of district hospital hospitalizations [19].

Several studies concentrated on the prevalence of hypertension in Rwanda, but none used the considerable mathematical statistical modeling approach, which can help decision-makers in taking short- and long-term measures; this is what we do in this study.

MMC and Bayesian statistics are two independent methods. The first method samples a distribution and the second is a theory of data interpretation, but the combination of the two is a powerful prediction technique. MCMC can be used in all Bayesian inferences and became a method of choice in data analysis and interpretation in almost all sciences [2]. It was very easy to predict the outcome given the cause but, with MCMC, the prediction of the cause, considering other causes and some other information, can help the prediction; this involved the calculation of conditional probability. MCMC is applied in many different fields. In quantitative health science, mathematical models are used to analyze and describe health processes.

The study used Gibbs sampling and the basic Markov Chain Monte Carlo method used in Bayesian statistics. Gibbs derives the posterior condition for each of the random variables and then simulates the posterior from the target joint posterior by iteratively sampling a value for a random variable from its corresponding posterior condition. All other variables are fixed to their existing values [20].

The Griddy Gibbs sampler, Gibbs sampling, or a Gibbs sampler is an MCMC algorithm used to obtain a sequence of observations, which are approximated from a specified multivariate probability distribution when direct sampling is difficult. In Gibbs sampling, all proposals are accepted with an acceptance probability equal to one [Yildirim]. A consecutive set of events in which the outcome at any given point depends on a constant probability is called a stochastic process [21].

Markov Chain Monte Carlo (MCMC) techniques are sampling methods derived from probability distributions using Markov chains. MCMC allows the users to approximate aspects of posterior distributions that cannot be directly calculated [22].

A Markov chain is completely characterized by defining the finite set of possible states S and the stationary probabilities of transition between these states [23].

The MCMC method was applied for sampling from the probability distribution by creating a Markov chain with a set of parameters that combine the parameter values representing the parameter distribution at the equilibrium distribution [24]. 

It is important to assess the problem of hypertension and its associated features. Health care providers need to be trained in this regard so that hypertension can be diagnosed and managed at primary care level and the complications of uncontrolled hypertension can be avoided [25,26]. The MCMC is a method which can help to understand the phenomena.

The rest of the article is organized as follows: Section 2 presents the materials and the methodology, Section 3 focuses on the results and the conclusion is presented in Section 4. 

## 2. Material and Methods

In the study of chronic diseases, decision trees (DT), support vector machines (SVM), artificial neural networks (ANN), linear regressions (LR), K-nearest neighbors (KNN), and naive Bayes (NB) are some machine learning techniques that were used and found to be successful. However, making predictions using these techniques is still a challenge. To introduce new knowledge and ideas, this study used the Markov Chain Monte Carlo (MCMC) method of prediction. A Markov Chain is a mathematical system that relies on state transitions according to a specific probability formula. Markov processes are used to explain and predict the processes that occur when a random variable of values changes over time, and each value depends solely on the state that comes before it, not on other previous states. The method employs various states, and a process can change between them with a specified transition probability. The future state of an MCMC process is independent of the past, as the present and the probability are constant. MCMC is employed in a wide range of applications. For instance, in medicine, it was proposed that the patient is always in one of the few discrete health states known as Markov states [2].

A transition matrix, which is a square matrix expressing the probability of moving from one state of a system to another state, can be determined by a particular method. 

If the transition probability is $p\left(n|m\right)=T^{mn}$, the transition matrix can be written as:

(1) $$T=\left(\begin{array}{c} T^{11}\;\;\;T^{12}\;\;\;T^{13}\ldots T^{1n}\\ T^{21}\;\;\;T^{22}\;\;\;T^{23}\ldots T^{2n}\\ .\\ .\\ .\\ T^{n1}\;\;\;\;T^{n2}\;\;\;\;T^{n3}\ldots T^{nn} \end{array}\right)$$

Element of the matrix satisfying $0\leq T^{mn}\leq 1$ and $\overset{n}{\underset{i=0}{\sum }}T^{mi}=1$.

The Gibbs sampling method, a specific type of Metropolis–Hasting method, is one of the methods used to calculate the elements of the probability transition matrix [27]. This approach makes use of the conditional distribution of one component of the state vector given the other components. The unique property of the Gibbs approach is its ability to be applied repeatedly without affecting the results of the initial state. The result of several iterations remains the same as the result of a single iteration. The Gibbs sampling method is used when it is difficult to sample from a joint probability density function, for example, $p\left(x_{i},x_{j}\right)$, but simpler to sample from the conditional distributions $p(x_{i}|x_{j})$ and $p\left(x_{j}|x_{i}\right),\;i\neq j$ for i=1, 2, 3…k and j=1, 2, 3, …k, where $p\left(x_{i}|x_{j}\right)\;$ is the posterior distribution of the parameter of interest $x_{i}$ and k is the number of states.

Before simulating the posterior from the target joint posterior using the Gibbs technique, each random variable’s posterior condition must be determined. This can be achieved by iteratively choosing a random variable’s value from its corresponding posterior condition, holding all other variables constant at their current values.

Before applying the Gibbs sampler, let us outline its algorithm as follows [28]:

Let *p*$\left(x_{i}|x_{j};\;j\;\neq \;i\right),\;for\;i\;,j=1,\;\ldots ,\;k,$ be the conditional distributions to sample from, and let x~y mean “distributed according to”. Let the initial values be $x_{1}^{\left(0\right)},\;x_{2}^{\left(0\right)},\;\ldots ,\;x_{k}^{\left(0\right)}$ at the initial time 0, and $x_{1}^{\left(t\right)},\;x_{2}^{\left(t\right)},\;\ldots ,\;x_{k}^{\left(t\right)}$ be the values at time t; then, the value at time t+1 depends only on the previous values. 

That is:

$$x_{1}^{\left(t+1\right)}\sim p\left(x_{1}|x_{2}^{\left(t\right)},\;x_{3}^{\left(t\right)},\ldots \;x_{k}^{\left(t\right)}\right),\;x_{2}^{\left(t+1\right)}\sim p(x_{2}|x_{1}^{t+1},\;x_{3}^{t},\ldots \;x_{k}^{t}),\;\vdots \;x_{i}^{\left(t+1\right)}\sim p\left(x_{i}|x_{1}^{\left(t+1\right)},\;x_{2}^{\left(t+1\right)},\;x_{i-1}^{\left(t+1\right)},\;x_{i+1}^{\left(t\right)},\ldots \;x_{k}^{\left(t\right)}\right),\;x_{k}^{\left(t+1\right)}\sim p\left(x_{k}|x_{1}^{\left(t+1\right)},\;x_{2}^{\left(t+1\right)},\;\;\;x_{i}^{\left(t+1\right)},\ldots \;x_{k-1}^{\left(t+1\right)}\right).$$

Since we need random values to serve as initials in Gibbs’ algorithm, the iterations may not necessarily depend on the actual posterior distribution. Therefore, MCMC ensures that the target joint posterior is determined from the stationary distribution of the samples generated by Gibbs’ algorithm. The conditional distribution is determined using Bayes’ rule [1,29]:

(2) $$P\left(B_{j}|A\right)=\frac{P\left(A|B_{j}\right)P\left(B_{j}\right)}{\sum_{j=1}^{n}P\left(A|B_{j}\right)P\left(B_{j}\right)}\;\;\;$$

where $P\left(A|B_{j}\right)$ is the posterior, $P(B_{j}|A)$ is the likelihood, and $P\left(A\right)$ is the prior.

Let X and Y be two random variables, and $\left(x_{0},y_{0}\right)$ be the initial point. Then, sample from the condition probability distribution of X given $Y=y_{0}$ and the next sample Y given $x_{1}$ in the previous sampling. Repeat the same up to n−1. Therefore, the algorithm is as follows:

1. Initial values $\left(x_{0},y_{0}\right);$
2. Sample: $x_{1}\sim p\left(x|y_{0}\right)\;i.e.,\;\left(X|Y=y_{0}\right);$
  State $\left(x_{1},y_{0}\right);$
  Sample: $y_{1}\sim p\left(y|x_{1}\right)\;i.e.,\;(Y|X=x_{1})$;
  Convert a random variable $\left(x_{1},y_{1}\right);$
3. Sample: $x_{2}\sim p\left(x|y_{1}\right)\;i.e.,\;(X|Y=y_{1};$
  State $\left(x_{2},y_{1}\right)$;
  Sample: $y_{2}\sim p\left(y|x_{2}\right)\;i.e.,\;\left(Y|X=x_{2}\right);$
  Convert a random variable $\left(x_{2},y_{2}\right);$
4. Repeat 2 and 3 as many times as k.

After repeating the process k times, a Gibbs sequence of length k was generated and the set $\left(x_{0},y_{0}\right)$, $\left(x_{1},y_{1}\right)$, $\left(x_{2},y_{2}\right)$, … $\left(x_{k},y_{k}\right)$, satisfied the property of being a Markov chain because the conditional distribution of $\left(x_{i},y_{i}\right)$ given in all previous pairs depended only on $\left(x_{i-1},y_{i-1}\right)$. The next section presents the results generated using these materials and this method.

## 3. Results

In [30], the researchers surveyed the data of 7240 people in 180 randomly selected villages, where multistage cluster sampling was used in the selection of the individuals. Using the results of this study, we defined the following states: 1—having hypertension; 2—tobacco users; 3—overweight; 4—obesity; 5—others. Let us assume that S=1, 2, 3,…n is the state space *S* = {1, 2, 3, 4, 5}, which is a sample space. This means that, for the Gibbs sampler, the parameter vector is divided into components 1, 2, 3, 4, 5. The elements of the transition matrix are calculated using (2) for *i*, *j* ∈ *S*.

The results in [30] showed that the prevalence of hypertension (state 1) was 15.3%, and the prevalence of tobacco use (state 2) was 26.2%. The prevalence of overweight (state 3) was 14.3%, the prevalence of obesity (state 4) was 2.8%, and the prevalence of others (state 5) was 41.7% [30]. These explain that $P\left(1\right)=0.153,\;P\left(2\right)=0.262,\;P\left(3\right)=0.143$, and $P\left(4\right)=0.028,\;\;\mathrm{and}\;P\left(5\right)=0.417$.

Using Bayes’ rule (2), we can obtain the following transition matrix when passing from one state to another.

$$P\left(1|2\right)=\frac{\left(P\left(1\right)P(2|1\right)}{P\left(1\right))P(2|1)+\left(P\left(1\right)\right)P(3|1)+\left(P\left(1\right)\right)P(4|1)+\left(P\left(1\right)\right)P(5|1)}=\frac{0.0393}{0.1257}=0.30823$$

$$P\left(2|1\right)=\frac{\left(P\left(2\right)P(1|2\right)}{P\left(2\right)P(1|2)+\left(P\left(1\right)\right)P(3|1)+\left(P\left(1\right)\right)P(4|1)+\left(P\left(1\right)\right)P(5|1)}=0.20325$$

and $p\left(1|3\right)=\frac{0.02145}{0.1275}=0.16823$.

Similarly, we calculate p(i|j) for i,j=1, 2, 3, 4, 5.

(3) $$T=\left(\begin{array}{ccccc} 0.00001 & 0.30823 & 0.16823 & 0.03294 & 0.49059\\ 0.20325 & 0.00001 & 0.19378 & 0.03796 & 0.565\\ 0.17503 & 0.30575 & 0 & 0.03264 & 0.48658\\ 0.1543 & 0.05546 & 0.14695 & 0.21419 & 0.4291\\ 0.25729 & 0.44938 & 0.24528 & 0.04804 & 0.00001 \end{array}\right)$$

From the result in the transition matrix, there was no absorbent state; all states communicated with each other. When a transition matrix was estimated using a given dataset, general information can be evaluated using the associated table.

Let $X^{\left(0\right)}=\left(0.153\;0.262\;0.143\;0.028\;0.417\right)$ be a starting point using the transition matrix in (1); let $X^{T}$ be the transpose of matrix X, the predicted point at time n, is given by the relation:

(4) $$X^{\left(n\right)}=T^{\left(n-1\right)}\times {\left(X^{\left(0\right)}\right)}^{T}.$$

Considering the transition matrix of a Markov chain, rows and columns cannot be interchanged; rows stand for previous observations and columns for present observations. The long-term probability of the system was found by powering the transition matrix, $T^{n}$ as n→∞; the entry in the row approached the long-term probability and the column weakly and monotonously increased. 

Several frameworks and packages can be used to determine the prevalence of an abnormal and human chronic disorder. We present numerical information about the prevalence and applied the necessary operations to find the results using MATLAB packages. Using the data in [1,18] and the transition matrix calculated in (3) as well as relation (4), Table 1 shows the annual prevalence of states in different years. The rows stand for hypertension, tobacco users, overweight, obesity, and others. 

**Figure 1:**
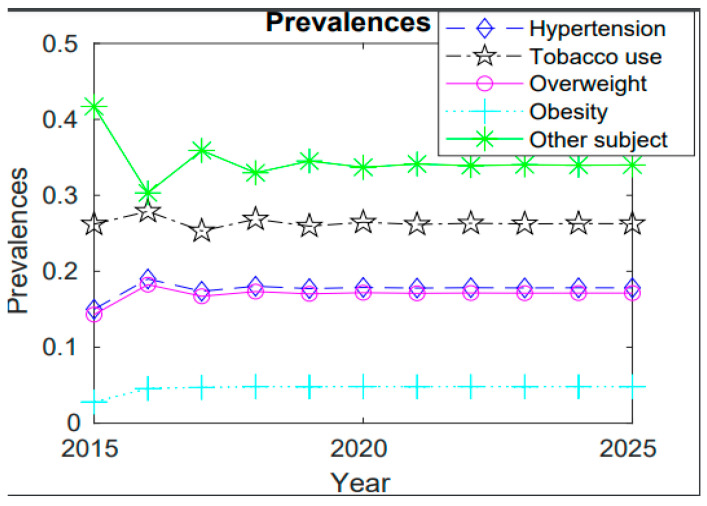
Prevalence of state from 2015 to 2025.

To analyze the trend of prevalence of hypertension, tobacco users, overweight, and obesity from 2015 to 2025, the numerical results are illustrated in Figure 1.

## 4. Conclusions

The increased prevalence of hypertension and associated risk factors in Rwanda might have negative social and health effects. Therefore, understanding hypertension’s tendencies and how these may affect public health is crucial to predict it. The knowledge presented in this article may be used to assist in alleviating societal and economic concerns by reducing healthcare costs and preventing diseases brought on by the country’s rise in hypertension.

According to the findings, the prevalence of hypertension is estimated to reach 17.82% in 2025, with tobacco use, being overweight or obese, and other risk factors having respective prevalences of 26.26%, 17.13%, 4.80%, and 33.99%. A higher decrease occurred in 2016–2017 of almost 19% and 17%, respectively, due to the efforts made by the Government of Rwanda (GoR) and stakeholders through various campaigns and physical activities, which established Rwanda a reference point: a champion of healthy lifestyle initiatives [18]. 

When the years 2015, 2020, and 2025 were compared, the year 2020 showed a high prevalence of 17.87%, since there was less knowledge about diseases and a change in lifestyle occurred due to the nation’s high level of development. However, in 2025, information may be made public through a variety of means, such as social media and health workers, and certain actions may be taken by internal and external stakeholders to prevent or slow the expected 17.82% increase in the prevalence of diseases. The prevalence of hypertension and overweight is decreasing at approximately the same rate; however, compared to previous years, the prevalence of obesity will remain high in 2025, indicating the need for special efforts to prevent obesity through engaging in physical activity, maintaining a healthy diet, and taking other preventative measures. It was found that education can contribute to a reduction in the prevalence of hypertension, as educated people can understand the associated risk factors and change their lifestyle. As a result, the widespread adoption of this policy will result in an excellent and noticeable decrease in NCDs, as it can serve as a bridge between citizens and health sensitizers [20]. 

Smoking, as one of the risk factors, had a prevalence of 26.2% in 2015; according to the results, this will reach 26.26% in 2025. The prevalence decreased from 27.89% to 25.31% between 2016 and 2017 and from 26.86% to 25.94% between 2018 and 2019 because of health sector actions and policies such as restrictions on tobacco sales to minors. The increase in cases could be attributed to environmental factors such as stress, inactivity, exposure to secondhand smoke, and poor diets that are high in salt and low in potassium.

While the prevalence of obesity as a significant risk factor for hypertension is expected to be high in 2025 compared to earlier years, the prevalence of both overweight and hypertension is rising at almost the same rate. This suggests that more efforts must be made to prevent obesity by exercising, eating a healthy diet, and other preventative measures.

Hypertension acts as a key barrier to the alleviation of poverty and to sustainable development; therefore, the government needs to prioritize the issues of diabetes and hypertension by showing the public that it they are serious, costly, and prevalent health problems, and they are on the national agenda. The country needs to design national strategies to tackle diabetes and hypertension as part of an incorporated approach to prevent and care for non-communicable diseases in general, as well as other major related risk factors. The health care system needs to be better supported to develop cost-effective services for the prevention and control of hypertension in primary health care settings. Lack of awareness in the population and a lack of detection and monitoring of hypertension could contribute to the increase in its prevalence.

At present, we wish to perform different operations in real time by developing appropriate uses of applied mathematics and statistics, focusing on concrete applications. Still, we must accumulate knowledge in different, appropriate branches of science and create models and relations to ensure a compact and adequate description when studying phenomena and processes to solve real-life problems. In other words, it is desirable to advance from abstract to concrete in every possible area. The use of Markov Chain Monte Carlo methods in medicine could make it possible to predict the prevalence of type 2 diabetes and hypertension in the future, and this would help us to take adequate measures for preventive work and planning. Data on hypertension in Rwanda are still limited. However, the prevalence and incidence of hypertension are increasing with rural-to-urban migration. Evidence shows that the cost of managing diabetes and hypertension is very high. In this study, hypertension in Rwanda was predicted using the Gibbs sampling method. The increasing prevalence of hypertension was found to be a result of a variety of factors; some of these can be controlled and others cannot. Although doctors, physicists, and other medical experts play a significant role in the prevention and treatment of this disease, there are also important public health issues that must be addressed. The government of Rwanda should establish short-, middle-, and long-term policies to prevent non-communicable diseases and reduce hypertension in the labor force to promote the long-term health of its population, while also reducing the prevalence of obesity in the country. Strengthening sports activities at all levels—from the individual to the professional—would help to achieve this. Regular checkups are advised around the country in both rural and urban areas, and nutritional experts should suggest and identify healthy foods that could help lead to a better quality of life. To reduce the risk of developing high blood pressure and its complications, precautions must be taken. Priority should be placed on nutrition, promoting a healthy lifestyle by reducing salt intake, eating fruit and vegetables, and reducing saturated and total fat intake, as well as controlling alcohol intake, encouraging regular physical activity to help children and young people to maintain a normal weight, stopping tobacco use and limiting exposure to tobacco products, and managing stress through meditation. This request is made to everyone, to ensure they are active in lowering blood pressure at all levels, from the individual to the international level. As the rate of hypertension continues to increase, as well as associated risk factors, preventative measures should be taken. The government could also use social media to raise awareness of these diseases through theater, comedy, dialogue, and discussion. This could be helpful because it will interest children and adults, mostly young adults, who will be a major part of society in the future. Health care professionals should test diabetes and hypertension in all persons above a certain age; this measure could be established by the Ministry of Health, even if patients are not showing any symptoms and should take place at least semiannually. Advice should be offered to patients with mild diabetes and hypertension on lifestyle modifications, such as reducing fat and salt intake, increasing physical activity, weight loss in people who are overweight, and a diet with an increase in fresh fruit and vegetables. There is a need for campaigns that promote the responsible consumption of alcohol and campaigns to avoid tobacco use. Since the cost of drugs and medicine is very high, patients should be empowered and motivated to join associations, so that they can undergo standard and proven treatment policies based on the most effective drugs, as recommended by the WHO in its model list of essential medicines in partnership with government and non-government organizations to ensure the consistent availability of insulin and other anti-diabetes drugs. This will strengthen the national capacity to collect and analyze data for both the health sector and researchers. Further research may focus on the awareness of the disease and its associated risk factors, as well as different preventive techniques.

## Figures and Tables

**Table 1:** Annual prevalence of states.

2015	2016	2017	2018	2019	2020	2021	2022	2023	2024	2025
0.1500	0.1899	0.1737	0.1803	0.1772	0.1787	0.1779	0.1784	0.1781	0.1783	0.1782
0.2620	0.02789	0.2531	0.2686	0.2594	0.2645	0.2617	0.2632	0.2624	0.2628	0.2626
0.1430	0.1824	0.1671	0.1733	0.1703	0.1718	0.1710	0.1714	0.1712	0.1713	0.1713
0.0280	0.0456	0.0471	0.0481	0.0479	0.0481	0.0480	0.0481	0.0480	0.0481	0.0480
0.4170	0.3032	0.3591	0.3297	0.3452	0.3369	0.3413	0.3390	0.3402	0.3396	0.33999

## Data Availability

This research used secondary data published in Republic of Rwanda (2015), Rwanda Non-Communicable Diseases Risk Factors Report.
